# Ventilation during extracorporeal membrane oxygenation for adult respiratory distress syndrome

**DOI:** 10.1186/cc13954

**Published:** 2014-06-30

**Authors:** Markus Kredel, Dominik Bierbaum, Christopher Lotz, Julian Kuestermann, Norbert Roewer, Ralf M Muellenbach

**Affiliations:** 1 Department of Anaesthesia and Critical Care, University of Würzburg, Oberdürrbacherstr. 6, 97080 Würzburg, Germany

## 

Lung protective ventilation is the mainstay of treatment in acute respiratory distress syndrome (ARDS). However, in patients who have severe ARDS and who are receiving extracorporeal membrane oxygenation (ECMO), the ventilation strategy might be modified. This was the subject of the review by Schmidt and colleagues [1] in a recent issue of *Critical Care*. We invited 39 ARDS centers in Germany to participate in a survey about respiratory and adjuvant ARDS therapy. The questionnaire was completed by 25 centers, 22 of which applied ECMO therapy at the specific time point of the survey (2011 to 2012).

The majority of the ARDS/ECMO centers preferred controlled mechanical ventilation during ECMO therapy, and only 14% favored assisted or spontaneous breathing. During ECMO, peak/plateau pressures and tidal volumes were lowered by 96% and 91% of the centers, respectively. The specified minimal and maximal tidal volumes were 2 (1.8 to 3) and 6 (4 to 6) mL/kg ideal body weight (median and interquartile range), respectively. Positive end-expiratory pressure (PEEP) was determined by using a ‘Best-PEEP-Trial’ in 50%, empirical means in 46%, or the ARDS-Network table in 36%. Pressure volume curves, computed tomography, and electrical impedance tomography, on the other hand, were rarely consulted to determine the optimal PEEP. However, a reduction in PEEP was done by only two institutions. Respiratory rates during ECMO were nearly equally distributed between fewer than 4, 4 to 12, 13 to 19, and 20 to 29 breaths per minute. The inspiratory-to-expiratory time ratios were 1:1 in seven and 1:2 in three centers. Inversed ratio (2:1 to 4:1) ventilation was conducted by 14%; only three applied recruitment maneuvers. Sixty-eight percent used 135° prone positioning, 36% full prone positioning, and 27% continuous axial rotation during ECMO therapy. Other strategies, such as the daily interruption of sedation and spontaneous breathing trials, were conducted in 72% and 86% of the centers, respectively. Eighteen used specific protocols for the weaning from ECMO. Early tracheostomy was aimed for in 64% and actually performed within the first 4 days of ECMO therapy in 41% of the centers.

The current survey shows that mechanical ventilation is frequently altered toward a protective, or even ‘ultraprotective’, strategy during ECMO. Adjuvant strategies were not limited by the presence of the extracorporeal circuit and remained integral elements of care. Notably, assisted or spontaneous breathing was seldom accomplished, despite daily interruption of sedation, the use of specific weaning protocols, and early tracheostomy. All in all, most centers relied on proven ARDS standards of care, assuming the physiologically most favorable response while randomized controlled trials during ECMO are missing.

## Abbreviations

ARDS: Acute respiratory distress syndrome; ECMO: Extracorporeal membrane oxygenation; PEEP: Positive end-expiratory pressure.

## Competing interests

The authors declare that they have no competing interests.

## References

[B1] Schmidt M Pellegrino V Combes A Scheinkestel C Cooper DJ Hodgson C Mechanical ventilation during extracorporeal membrane oxygenation Crit Care 2014 18 203 10.1186/cc13702 24447458 PMC4057516

